# Implementation and Evaluation of a Wiki Involving Multiple Stakeholders Including Patients in the Promotion of Best Practices in Trauma Care: The WikiTrauma Interrupted Time Series Protocol

**DOI:** 10.2196/resprot.4024

**Published:** 2015-02-19

**Authors:** Patrick M Archambault, Alexis F Turgeon, Holly O Witteman, François Lauzier, Lynne Moore, François Lamontagne, Tanya Horsley, Marie-Pierre Gagnon, Arnaud Droit, Matthew Weiss, Sébastien Tremblay, Jean Lachaine, Natalie Le Sage, Marcel Émond, Simon Berthelot, Ariane Plaisance, Jean Lapointe, Tarek Razek, Tom H van de Belt, Kevin Brand, Mélanie Bérubé, Julien Clément, Francisco Jose Grajales III, Gunther Eysenbach, Craig Kuziemsky, Debbie Friedman, Eddy Lang, John Muscedere, Sandro Rizoli, Derek J Roberts, Damon C Scales, Tasnim Sinuff, Henry T Stelfox, Isabelle Gagnon, Christian Chabot, Richard Grenier, France Légaré, Canadian Critical Care Trials Group

**Affiliations:** ^1^Département de médecine familiale et médecine d'urgenceUniversité LavalQuébec, QCCanada; ^2^Centre de santé et de services sociaux Alphonse-Desjardins (Centre hospitalier affilié universitaire de Lévis)Lévis, QCCanada; ^3^Division de soins intensifsUniversité LavalQuébec, QCCanada; ^4^Centre de recherche du CHU de Québec, Axe Santé des populations - Pratiques optimales en santé, Traumatologie – Urgence – Soins IntensifsQuébec, QCCanada; ^5^Département de médecine familiale et médecine d'urgenceUniversité LavalQuébec, QCCanada; ^6^Vice-décanat à la pédagogie et au développement professionnel continuFaculté de médecineUniversité LavalQuébec, QCCanada; ^7^Département de médecineUniversité LavalQuébec, QCCanada; ^8^Department of Social and Preventative MedicineUniversité LavalQuébec, QCCanada; ^9^Centre de Recherche du CHU de SherbrookeCentre Hospitalier Universitaire de SherbrookeUniversité de SherbrookeSherbrooke, QCCanada; ^10^Research UnitRoyal College of Physicians and Surgeons of CanadaOttawa, ONCanada; ^11^Faculté des sciences infirmièresUniversité LavalQuébec, QCCanada; ^12^Département Médecine MoléculaireCentre de recherche du CHU de QuébecUniversité LavalQuébec, QCCanada; ^13^Department of PediatricsUniversité LavalQuébec, QCCanada; ^14^École de psychologieUniversité LavalQuébec, QCCanada; ^15^Faculté de pharmacieUniversité de MontréalMontreal, QCCanada; ^16^Institut national d'excellence en santé et services sociauxMontréal, QCCanada; ^17^Adult Trauma ProgramMcGill University Health CenterMcGill UniversityMontreal, QCCanada; ^18^Radboud REshape Innovation CentreRadboud University Medical CentreNijmegenNetherlands; ^19^Telfer School of ManagementUniversity of OttawaOttawa, ONCanada; ^20^Hôpital du Sacré-Coeur de MontréalMontréal, QCCanada; ^21^Programme de traumatologieCHU de QuébecUniversité LavalQuébec, QCCanada; ^22^eHealth Strategy OfficeFaculty of MedicineUniversity of British ColumbiaVancouver, BCCanada; ^23^Centre for Global EHealth Innovation & Techna InstituteUniversity Health NetworkToronto, ONCanada; ^24^Institute for Health Policy, Management, and EvaluationUniversity of TorontoToronto, ONCanada; ^25^JMIR Publications IncToronto, ONCanada; ^26^Department of PediatricsFaculty of MedicineMcGill UniversityMontreal, QCCanada; ^27^Pediatric and Adolescent Trauma ProgramsMontreal Children's HospitalMcGill University Health CentreMontreal, QCCanada; ^28^Canadian Hospitals Injury Reporting and Prevention ProgramMontreal, QCCanada; ^29^Department of Emergency MedicineUniversity of CalgaryCalgary, ABCanada; ^30^Department of Critical Care MedicineQueen's UniversityKingston, ONCanada; ^31^Trauma ProgramSt. Michael's HospitalUniversity of TorontoToronto, ONCanada; ^32^Department of SurgeryUniversity of CalgaryCalgary, ABCanada; ^33^Department of Community Health SciencesUniversity of CalgaryCalgary, ABCanada; ^34^Interdepartmental Division of Critical Care MedicineFaculty of MedicineUniversity of TorontoToronto, ONCanada; ^35^Department of Critical Care MedicineSunnybrook Health Sciences CentreToronto, ONCanada; ^36^Department of Critical Care MedicineUniversity of CalgaryCalgary, ABCanada; ^37^School of Physical and Occupational TherapyMcGill UniversityMontreal, QCCanada; ^38^Table de consultationRéseau québécois de cardiologie tertiaireQuébec, QCCanada; ^39^Thales Research and TechnologyThales CanadaQuébec, QCCanada; ^40^Canadian Critical Care Trials GroupMontreal, QCCanada

**Keywords:** interrupted time series, wiki, quality improvement, knowledge translation, trauma care, stakeholder engagement, adapting knowledge tools

## Abstract

**Background:**

Trauma is the most common cause of mortality among people between the ages of 1 and 45 years, costing Canadians 19.8 billion dollars a year (2004 data), yet half of all patients with major traumatic injuries do not receive evidence-based care, and significant regional variation in the quality of care across Canada exists. Accordingly, our goal is to lead a research project in which stakeholders themselves will adapt evidence-based trauma care knowledge tools to their own varied institutional contexts and cultures. We will do this by developing and assessing the combined impact of WikiTrauma, a free collaborative database of clinical decision support tools, and Wiki101, a training course teaching participants how to use WikiTrauma. WikiTrauma has the potential to ensure that all stakeholders (eg, patients, clinicians, and decision makers) can all contribute to, and benefit from, evidence-based clinical knowledge about trauma care that is tailored to their own needs and clinical setting.

**Objective:**

Our main objective will be to study the combined effect of WikiTrauma and Wiki101 on the quality of care in four trauma centers in Quebec.

**Methods:**

First, we will pilot-test the wiki with potential users to create a version ready to test in practice. A rapid, iterative prototyping process with 15 health professionals from nonparticipating centers will allow us to identify and resolve usability issues prior to finalizing the definitive version for the interrupted time series. Second, we will conduct an interrupted time series to measure the impact of our combined intervention on the quality of care in four trauma centers that will be selected—one level I, one level II, and two level III centers. Participants will be health care professionals working in the selected trauma centers. Also, five patient representatives will be recruited to participate in the creation of knowledge tools destined for their use (eg, handouts). All participants will be invited to complete the Wiki101 training and then use, and contribute to, WikiTrauma for 12 months. The primary outcome will be the change over time of a validated, composite, performance indicator score based on 15 process performance indicators found in the Quebec Trauma Registry.

**Results:**

This project was funded in November 2014 by the Canadian Medical Protective Association. We expect to start this trial in early 2015 and preliminary results should be available in June 2016. Two trauma centers have already agreed to participate and two more will be recruited in the next months.

**Conclusions:**

We expect that this study will add important and unique evidence about the effectiveness, safety, and cost savings of using collaborative platforms to adapt knowledge implementation tools across jurisdictions.

## Introduction

### The Research Question

#### What Is the Problem to Be Addressed?

Injuries represent a major health and economic burden for Canadians. They are the most common cause of mortality for people between the ages of 1 and 45 years [1], costing Canadians 19.8 billion dollars in 2004 in direct and indirect costs [2,3]. Up to half of all patients with major traumatic injuries do not receive evidence-based recommended care [4-8]. A recent study conducted in partnership with the Institut national d'excellence en santé et services sociaux (INESSS) and funded by the Canadian Health Services Research Foundation [5] determined that many trauma practices in Quebec’s trauma centers are substandard because they underuse proven therapies [4,5]. Studies in several other countries have identified adverse events, including death, that occur in trauma centers because of their failure to adopt best practices [9-13]. Aside from underusing proven therapies, there is also evidence of overuse of diagnostic procedures with known side effects, such as full-body computerized tomography (CT) scanning that exposes patients to unnecessary ionizing radiation that may increase the risk of cancer [14,15]. An estimated one million children every year in the US are unnecessarily imaged with CT [16].

Promoting best practices in trauma care has become an urgent and strategic investment for the health of Canadians and others [17,18]. Unfortunately, the implementation of best practices in the chaotic, acute trauma care environment is a difficult task because of three main factors, which are (1) macroenvironmental (eg, lack of financial resources), (2) organizational (eg, unclear definition of responsibilities within trauma team), and (3) professional (eg, resistance to clinical guidelines) [19]. Studying strategies used to implement best practices in trauma care, the Commonwealth Fund study [19] identified that trauma systems needed better knowledge management and coordination of care through well-implemented guidelines (ie, recommendations about what to do), protocols (ie, detailed procedures for how to administer care), and pathways (ie, frameworks for organizing who administers care and why). Moreover, these tools must be flexible and responsive to individual patients and to accumulating bodies of evidence. Many different health organizations have, therefore, started using wikis to manage knowledge and coordinate care [20-28]. Increasingly popular among health professionals [29-32], wikis are websites based on a novel technology that allow people to view and edit the website’s content, with viewing and editing privileges determined by different levels of access. Wikipedia—the best-known wiki—has 365 million visitors per month, is the sixth-most popular website in the world and its medical articles, available in 271 languages, are viewed about 150 million times per month [33].

In partnership with our team of researchers, the INESSS in Quebec is exploring how wikis could be used to improve the delivery of care to trauma patients. The INESSS oversees the quality of trauma care in the province of Quebec, Canada. It is also the accreditation body that designates different trauma center levels. This organization has expressed the need to explore wikis as a solution to improve the quality of care in trauma. To this end, we have conducted a scoping review that found that wikis could be effective in supporting the implementation of best practices in health care [34-39]. We also conducted a survey that identified that trauma professionals are willing to use wikis and that they share many positive beliefs about using them [40]. Specifically, wikis could serve as centralized knowledge management systems helping clinicians and decision makers coordinate the implementation of best practices by collaboratively building knowledge translation (KT) tools (eg, guidelines, protocols, pathways, and patient decision aids) that meet their needs [41-45] and monitor their use using novel Web metrics [46,47]. Wikis’ other interesting features included their low cost [40,48-51], their broad and global availability [33], their adaptability to local practices, and their capacity to empower stakeholders [52-54]. Wikis were perceived to facilitate the sharing and updating of KT tools by different professionals and to help clinicians working in rural areas where access to specialized care is limited [55,56].

**Figure 1:**
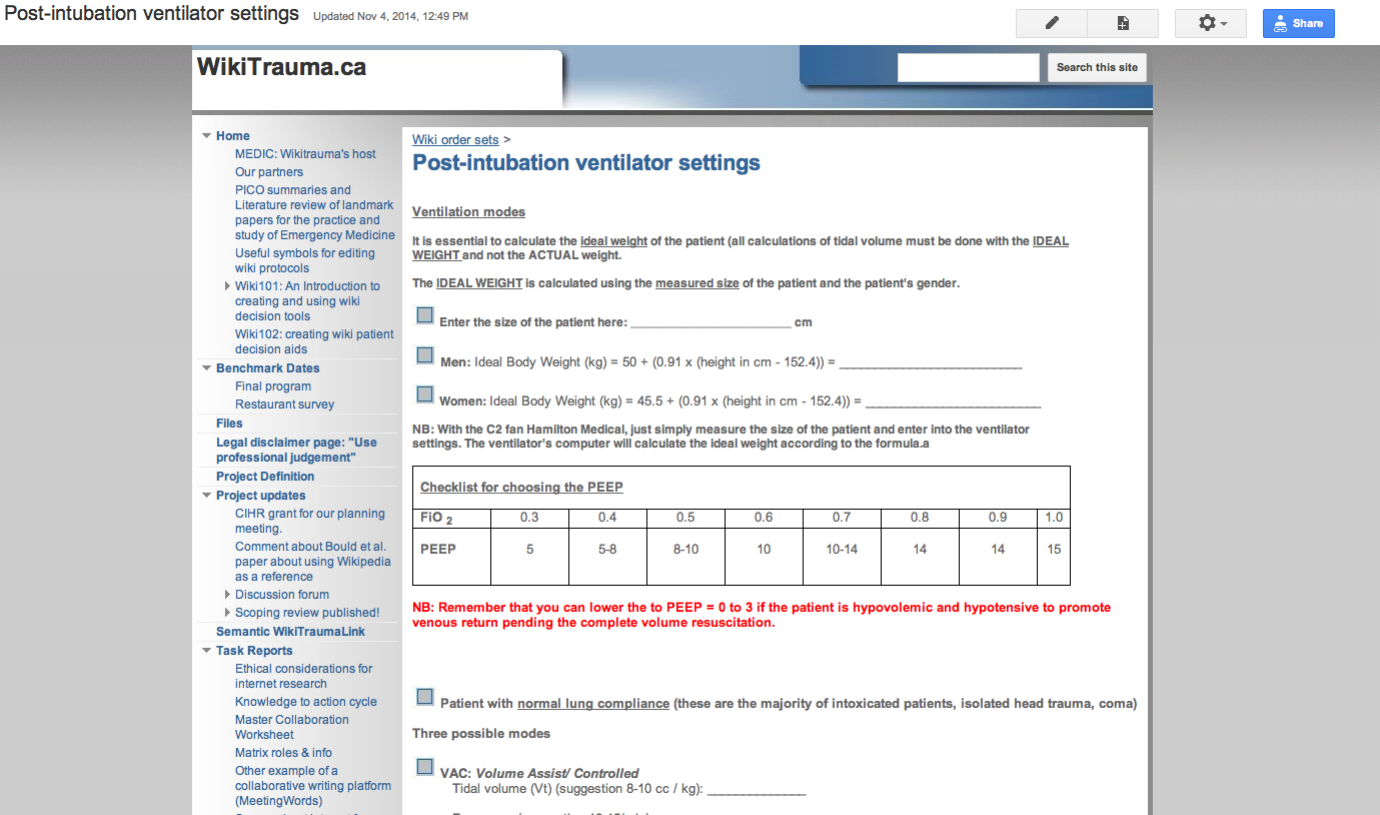
Screenshot of WikiTrauma order set.

**Figure 2:**
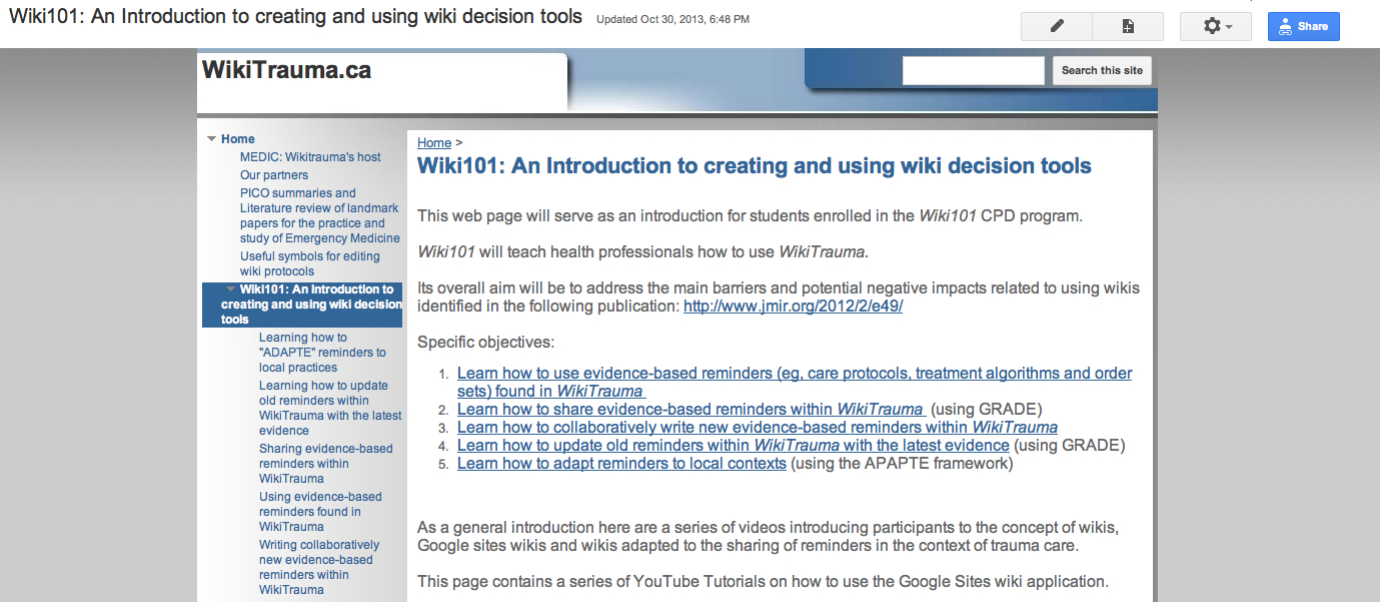
Screenshot of Wiki101 training program.

Building on these results, we held a meeting funded by the Canadian Institutes of Health Research (CIHR) in May 2014 in partnership with the INESSS, the Trauma Association of Canada, and the Royal College of Physicians and Surgeons of Canada to plan a study to evaluate WikiTrauma (see Figure 1), a wiki we created to promote best practices in trauma care. At this planning meeting we decided to study its implementation in a limited number of Quebec trauma centers as a first trial for this novel intervention. We also decided to create Wiki101 (see Figure 2), a theory-based continuing professional development (CPD) program, that will train participants at the selected trauma centers to use WikiTrauma effectively and safely in order to create and share different types of KT tools (eg, care protocols, order sets, and patient decision aids).

Thus, in partnership with the INESSS and in collaboration with our other WikiTrauma partners, we propose an interrupted time series to measure the combined effect of Wiki101 and WikiTrauma on the quality of trauma care in four trauma centers in Quebec. We also propose to conduct a mixed-methods process evaluation in parallel with this trial to explore possible causal mechanisms about how our combined intervention succeeds—or fails—to lead to improved quality of trauma care.

#### What Are the Principal Research Questions to Be Addressed?

**Figure 3:**
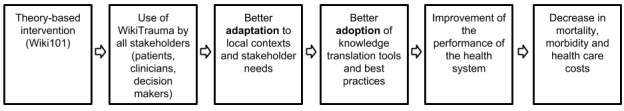
Conceptual framework underlying the proposed mechanism of action for the intervention.

Ultimately, we seek to test our hypothesis that our theory-based intervention (Wiki101) in combination with the use of WikiTrauma will result in better adoption of best practices in trauma care in Canada (see the conceptual framework in Figure 3), safer care (ie, fewer complications), improved patient outcomes, and less costly care.

#### Why Is This Project Needed Now?

Getting new evidence into health care practice is a slow and challenging process [57-65]. There is an ongoing and urgent need to find effective and low-cost methods of promoting best practices in all areas of health care [65-67] and, particularly, into interprofessional settings such as trauma care [17,68-72]. Recognizing that wikis capitalize on the free and open access to information, scientists, opinion leaders, and patient advocates have called for more research to determine whether wikis can equip decision-making constituencies to improve the delivery of health care [33,73], decrease its cost [49,74], and improve access to knowledge within developing countries [33,75-77]. Moreover, wikis are increasingly being used in health care by different academic institutions [32,41,78-81], health organizations [20,22,25,82,83], and health professionals [84-86] to share and disseminate information. As well, the principal knowledge user involved in our project (INESSS) is planning to use a wiki to promote best practices in trauma care, but would like to have more evidence about their use. Our CIHR-funded scoping review [34] confirmed that wikis have tremendous potential for improving the delivery of health care, but that a rigorous prospective trial to evaluate their effectiveness at implementing best practices is outstanding. Both our review and our survey identified an important need to test a theory-based approach addressing the main barriers that are preventing wikis from widely benefiting our health care system. The barriers most frequently mentioned, in order of frequency, were unfamiliarity with wikis, time constraints, lack of self-efficacy (ie, belief in one’s competence to use a wiki), and lack of access to a useful wiki containing reliable information for bedside decision making. For these reasons, we have designed WikiTrauma—a wiki promoting best practices—and Wiki101—a theory-based intervention—to maximize the potential benefits, to address the main barriers, and prevent any potential negative impacts of using a wiki to promote best practices in trauma care. In summary, there is sufficient evidence to support the conduct of this prospective interrupted time series for testing our novel intervention, which will combine a wiki to promote best practices in trauma care and a theory-based implementation strategy designed to maximize its benefit. This trial will inform our knowledge users about the impact of the combined effect of WikiTrauma and Wiki101 on the implementation of best practices in trauma care.

#### Best Practices in Trauma Care

##### Barriers to Implementing Best Practices in Trauma Care

Various aspects of trauma care can impede best practices [13]. Trauma professionals must often make quick decisions, mostly based on intuitive reasoning [87], which is fast, impulsive, effortless, and reflexive. While this serves trauma care well, it is also prone to error. Reminders (eg, care protocols) are knowledge tools [88] that improve intuitive decision making [87]. A recent systematic review indicated that noncomputerized reminders had the potential to improve practices in critical care [70]. Computerized reminders and clinical decision support systems, which were excluded from the previous review, offer different KT opportunities in trauma centers' hectic environments [89-91].

##### Systematic Reviews About Computerized Decision Support Systems and Barriers to Their Adoption

Systematic reviews indicate that computer-based reminders are effective interventions for fostering best practices in a variety of clinical areas [26,92-100], including in acute care [89]. Such reminders range from simple prescribing alerts to more sophisticated computer systems that support decision making. This said, health professionals have rejected many computer-based reminder systems on the grounds that they are slow, incompatible with work processes, unable to adapt to local practices, difficult to access, and/or are costly to implement [90,91,101-105]. Finding innovative ways to involve end users in designing, implementing, and evaluating reminder systems is key to increasing their use and their impact on health outcomes. Novel collaborative applications like wikis offer an easy and inexpensive solution [31].

##### Theoretical Framework Supporting the Use of Wikis as a Driver for Change in Health Systems

According to behavior-change theories, self-efficacy—roughly defined as an individual’s belief in his/her own competence—is one of the most important cognitive determinants of behavior [106-109]. By involving health professionals in sharing, updating, and creating practical reminders, wikis—highly accessible, interactive vehicles of communication—have the potential to increase professionals’ self-efficacy in using reminders [29,30,74].

##### Rising Use of Wikis in the Health Care System

Studies have found that 70% of junior physicians (mostly residents) use Wikipedia weekly [84], that 50-70% of physicians use it as a source of information in providing care [33], and that 35% of pharmacists refer to it for drug information [85]. Different large health care organizations (eg, Canadian Agency for Drugs and Technologies in Health [110-112], US National Institutes of Health [20,113], The Cochrane Collaboration [22], World Health Organization [83], and several universities [32,41,78-81,114]) are exploring the use of wikis and/or Wikipedia for different purposes. There is a rising use of wikis in health care and, consequently, increased potential safety risks involved with using nonvalidated information for the care of patients. Therefore, we believe there is an urgent need to evaluate the positive benefits wikis could provide in improving the quality of care, while limiting the potential negative effects. We intend to do this by conducting a rigorous and well-planned prospective trial in the controlled setting of a closed wiki (WikiTrauma) managed by strong central leadership (INESSS).

### Objectives

Our main objective will be to study the combined effect of WikiTrauma and Wiki101 on the quality of care in four trauma centers in Quebec using an interrupted time series design. Our secondary objectives will be (1) to evaluate the impact of our intervention on mortality, rate of complications, length of stay, and the Functional Independence Measure (FIM), (2) to evaluate participants' opinions about the combined intervention—Wiki101 and WikiTrauma, (3) to evaluate the quality of the different knowledge tools developed in WikiTrauma, and (4) to estimate the costs saved by sharing the different knowledge tools within WikiTrauma.

## Methods

### Pilot-Testing of WikiTrauma and Wiki101 Before the Prospective Trial

In consultation with two human factors specialists (HW, ST) and using the versions of WikiTrauma and Wiki101 developed at the planning meeting, we will further refine WikiTrauma and Wiki101 by employing user-centered design methods focused on our users' needs [115,116]. A rapid, iterative prototyping process with 15 health professionals from nonparticipating centers will allow us to efficiently identify and resolve usability issues prior to finalizing the definitive version of WikiTrauma and Wiki101 for the interrupted time series [117,118].

### What Is the Proposed Trial Design?

**Figure 4:**
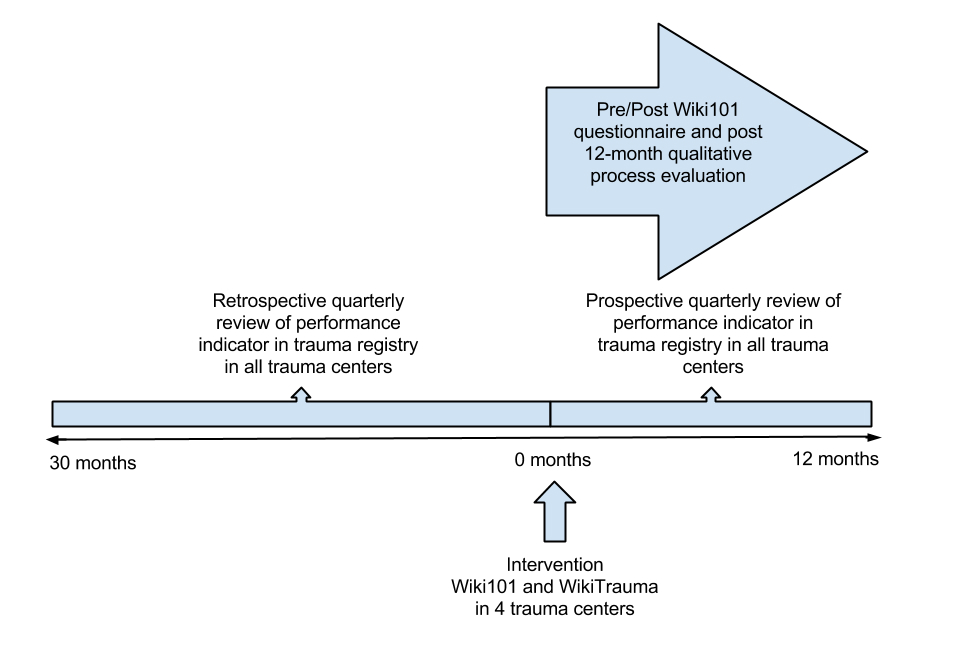
Diagram representing the interrupted times series design.

This study will be an interrupted time series with a parallel, theory-based process evaluation alongside the trial (see Figure 4). In the context of quality improvement, the interrupted time series is a simple but powerful tool used for evaluating the impact of a quality improvement program [119]. Our time series—repeated observations of the quality of care collected over time—will be divided into two segments. The first segment will comprise 10 retrospective, quarterly measurements of the quality of care measured before our intervention (a period of 30 months), and the second segment will be four prospective, quarterly quality of care measurements after our intervention (12 months). There are 57 adult-designated trauma centers of varying levels in Quebec—three level I, 26 level II, and 28 level III trauma centers. Four trauma centers will be selected in Université Laval's trauma network. We already identified one level I trauma center and a level II trauma center as participants. We will recruit two level III centers to complete our targeted sample of participating centers. Participants will not be blinded to their study assignment, however, all analyses will be blinded. The control group will comprise all of the 53 remaining adult trauma centers in the province.

### What Are the Planned Trial Interventions?

#### Experimental Group

Participants from trauma centers assigned to the experimental group will receive a password to access and complete the Wiki101 course and to use WikiTrauma. They will receive three reminders at 2-week intervals to complete Wiki101. Before and after each Wiki101 course, participants will be administered a validated questionnaire to measure changes in opinion and beliefs about using WikiTrauma. Questionnaires will also be repeated after the prospective 12-month period. After each course, participants will also receive a 2-week reminder about skills taught during the course.

#### Control Group

Participants in the control group will receive an email promoting access to the regular INESSS webpage and will also receive three reminders at 2-week intervals to access the website. They will not have access to view or edit WikiTrauma.

#### Management of WikiTrauma During the Trial

For the purpose of this trial, to monitor its use, and ensure its quality, access to WikiTrauma will be protected by password.

#### Quality of Information Monitoring

Since the wiki content can be constantly changed by the participants, the quality of information and the strength of evidence will be assessed weekly by a medical expert (JL) and monthly by the steering committee using a standardized evaluation form. This committee will edit any serious deviations from recognized standards of care and will flag controversial topics to stimulate discussion within the wiki community.

### What Are the Proposed Practical Arrangements for Allocating Participants to Trial Groups?

All professionals and decision makers working in the four participating trauma centers will be eligible to participate. With the help of the local leaders on the trauma committee, we will recruit as many clinicians (eg, physicians, nurses, respiratory therapists, and pharmacists) and decision makers (eg, heads of Emergency Department, Surgery Department, and Critical Care Department) as possible. All participating trauma committees will also be asked to designate five patient representatives to take part in the construction of various tools designed for their use (eg, decision aids and patient handouts). In each center, we will present the project to the local representatives of each trauma committee. We will provide a hands-on Wiki101 session to all the members of the local trauma committee and the five patient representatives, who will then become the local leaders able to teach their colleagues how to access the wiki and contribute to its content. An online version of Wiki101 will be available to all clinicians and patient representatives for future consultation.

### What Are the Proposed Methods for Protecting Against Sources of Bias?

Although blinding of the participants and randomization are not feasible in this small trial, we will mobilize all efforts to minimize any other sources of bias. All data collectors (medical archivists) will be blinded to the allocation group. Throughout our study, we will prevent contamination by protecting Wiki101 and WikiTrauma by password and note any potential competing intervention. We will also identify any professional working in more than one participating trauma center to consider the impact of this potential source of bias. Wiki101 will be a standardized online training program. We will encourage all participants to complete all 12 months of the study. To minimize a potential Hawthorne effect, our control group will receive an invitation to consult the INESSS website at the beginning of the study. Moreover, our proposed study design of an interrupted time series provides the advantage of controlling for secular trends in the data.

### What Are the Planned Inclusion/Exclusion Criteria?

#### Inclusion Criteria

We will select four trauma centers—one level I, one level II, and two level III trauma centers. The two level III centers will be identified by the authors based on their willingness to participate and collaborate with the other trauma centers for the 12-month project. At the individual level, study participants must be decision makers (eg, trauma program coordinators) or health care professionals (eg, emergency physicians, critical care physicians, trauma surgeons, nurses, respiratory therapists, physiotherapists, or pharmacists). Patient representatives will be selected without any restrictions or limitations with regard to their qualifications. These patient representatives could also be caregivers to existing trauma patients. Health care professional students and trainees (eg, residents, medical students, and nursing students) will have the same access to WikiTrauma and Wiki101 as fully certified professionals.

#### Exclusion Criteria

At the cluster level, a trauma center will not be eligible to participate if more than 50% of the members of the local trauma committee refuse to participate. Reasons for exclusion or refusal to participate will be documented. Pediatric trauma centers will be excluded.

### What Is the Proposed Duration of the Treatment Period?

Wiki101 will take 3 hours to complete for each participant and they will have access to use Wiki101 and WikiTrauma for 12 months.

### What Is the Proposed Frequency and Duration of Follow-Up?

Aside from our pre- and post-Wiki101 questionnaire and the 2-week reminder after completing Wiki101, we will only administer a final questionnaire after the 12-month treatment period.

### What Are the Proposed Primary and Secondary Outcome Measures?

The primary outcome measure will be the change over time in a validated, composite performance indicator score based on 15 process performance indicators found in the Quebec Trauma Registry [4]. The secondary outcome measures will be rates of complications, length of stay, mortality, and the FIM. These will also be found in the Quebec Trauma Registry. Other secondary outcome measures will be the following: (1) intention to use WikiTrauma and the sociocognitive determinants of this intention, (2) the self-reported use of WikiTrauma in clinical practice, (3) the actual frequency of WikiTrauma use—number of visits, length of visits, number of visitors, and number of unique visitors, (4) the quality of information contained within WikiTrauma, (5) the frequency of content modifications—number of visitors having modified content, number of pages modified, number of new pages created, and number of pages having generated an edit war, (6) participants’ comments about what worked and improvements suggested, (7) the estimated annual cost of maintaining WikiTrauma, (8) the cost of delivering Wiki101, (9) the estimated cost of creating new knowledge-decision tools, and (10) the estimated cost of updating old knowledge-decision tools.

### How Will the Outcome Measures Be Measured at Follow-Up?

We will measure quarterly composite performance scores from the Quebec Trauma Registry for all 57 adult trauma centers. Data in the Quebec Trauma Registry is routinely collected in all Quebec trauma centers every 3 months. The composite performance score is calculated as the average of 15 other indicators routinely collected in the Quebec Trauma Registry [4]. This score has good discrimination, construct validity, criterion predictive validity, and forecasting properties [120]. Mortality rates, complication rates (for delirium, pneumonia, and deep venous thrombosis), length of stay, and FIM will also be measured on a quarterly basis for all 57 trauma centers from routinely collected data in the Quebec Trauma Registry. The intention to use WikiTrauma will be measured by a validated questionnaire [40]. The actual wiki use and the frequency of content modification will be measured on a quarterly basis using a Google Analytics account linked to WikiTrauma.

### Safety Monitoring and Quality Assurance

Since participants can change wiki content, the quality of information and the strength of evidence will be assessed weekly by an INESSS medical expert, and monthly by the scientific committee using a standardized evaluation form. This committee will edit any serious deviations from recognized standards of care and will flag controversial topics to stimulate discussion within the wiki community. The quality of different KT tools will be evaluated using the Grading of Recommendations, Assessment, Development and Evaluation (GRADE) methodology for grading quality of evidence and strength of recommendations [121]. To estimate the amount of supervision that was needed by the medical supervisor at INESSS, we will document the number of pages modified and created, and the number of edit wars. In order to ensure that only high-quality and officially approved knowledge tools will be used in clinical practice, wiki pages that are not approved for clinical use by local trauma committees will be color-coded in RED with a warning message to say that the page is currently under construction. Pages that are approved by local trauma committees will be color-coded in GREEN for use only in the trauma center that approved the page. To estimate activity that was generated by our wiki and the amount of supervision that was needed during this trial by the medical supervisor at INESSS, we will study the wiki’s revision history page to document the number of visitors having modified the content, the number of pages modified, the number of new pages created, and the number of pages having generated an edit war [122] on a quarterly basis. An edit war will be defined as more than three reverts by a single editor on a single page within a 24-hour period. An edit that undoes other editors' actions will count as a revert. All cases of potential patient harm reported by any quality assurance committee or participant will be described and declared.

### Sample Size

As a rule of thumb for an interrupted time series, 10 measurement points before and 10 measurements after an intervention provides 80% power to detect a change in level of 5 standard deviations (of the predata) only if the autocorrelation is greater than .4 (ie, extent to which data collected close together in time are correlated with each other) [123]. In our case, we will be able to measure 10 measurement points before (30 months), but the period of observation after our intervention will be limited to 12 months—4 quarters is equal to 4 measurement points. This will decrease our power, but we are currently applying for funding from other sources to collect data for a total of 32 postintervention months (10 quarters).

### Data Analysis

Segmented regression will be used to measure, statistically, the changes in level and slope in the postintervention period compared to the preintervention period [119]. Thus, we will present a regression model with different intercept and slope coefficients for the pre- and postintervention time periods. We will compare the changes in quality of care measured at our four intervention trauma centers to the changes in quality of care measured at the other 53 trauma centers where no experimental intervention occurred. During the implementation period of WikiTrauma, we will continue to measure the impact on the quality of care. However, we will only proceed to compare the change in slope in the postintervention period once WikiTrauma has been fully implemented. We will use a Durbin-Watson test to verify the presence of autocorrelation and use an autoregressive error model to correct for this serial correlation.

### Qualitative Content Analysis

We plan to enlist two researchers experienced in qualitative content analysis who will review participants' written questionnaire answers to identify the barriers in using our intervention. They will also try to understand how our combined intervention succeeded—or failed—to lead to improved quality of trauma care. When consensus between the two reviewers is not possible, a third reviewer will be consulted.

### Study Duration

This project is planned to last 18 months. We have planned 3 months to implement the trial, including ethics approval in the four designated trauma centers and for delivering Wiki101 to the four local trauma committees. We will analyze all retrospective data obtained from the Quebec Trauma Registry in the first 3 months of our study and every 3 months thereafter for a total of 12 months. The last 3 months will be used to prepare our datasets, conduct our various analyses, and write our final report.

### Ethical Considerations

We will apply for ethical approval to conduct this trial in all four participating trauma centers. All participants will be asked to consent before accessing the wiki for the first time and before any questionnaire administration. Local trauma committees will be consulted and we will obtain approval and support from each trauma center's chief executive officer. Patient participants will also be asked to complete a consent form before participating in any phase of this trial.

A legal disclaimer will also be posted on the wiki site asking participants to always use their clinical judgement first. Clinical judgement should never be replaced by any information found in a protocol based in WikiTrauma. In addition, clinicians should only use the wiki pages that have been approved by their local trauma committee.

All personal information on study participants will remain anonymous and we will not publish the names of any of the participating trauma centers. All sensitive information will be kept in a locked filing cabinet at the principal investigator’s (PI) research center or in a password-protected computer at the research center.

## Results

This project was funded in November 2014 by the Canadian Medical Protective Association. We expect to start this trial in early 2015 and preliminary results should be available in June 2016. Two trauma centers have already agreed to participate and two more will be recruited in the next months.

## Discussion

We expect that this study will yield important and unique evidence about the effectiveness, safety, and cost savings of using collaborative platforms to adapt knowledge-implementation tools across jurisdictions. A recent scoping review had not identified any prospective studies analyzing the impact of a wiki intervention on the quality of care in any field of health care [34]. Thus, to the best of our knowledge, this will be the first interrupted time series evaluating the impact of a wiki on the implementation of best practices in trauma care. Patient safety science will gain from this project because we will investigate how WikiTrauma can help standardize care across our trauma system. This will be done by providing a unique collaborative tool that allows centers to learn from others in the implementation of evidence-based knowledge translation tools (eg, care protocols, order sets, and patient decision aids). WikiTrauma will also offer a unique knowledge-management platform to support the central leadership provided by provincial decision makers, such as the Institut national d'excellence en santé et services sociaux. This study will also provide a new platform for effective local collaboration between professionals, decision makers, and patients. Public- and patient-involvement programs will gain insight about using wikis to engage patients and the public in the implementation of best practices. Interprofessional education and quality-improvement programs will also learn about how these novel platforms can support collaboration and coordination in the implementation of novel best practices.

## Abbreviations

CHAU: Centre hospitalier affilié universitaire
CHU: Centre hospitalier universitaire
CIHR: Canadian Institutes of Health Research
CPD: continuing professional development
CSSS: Centre de santé et services sociaux
CT: computerized tomography
FIM: Functional Independence Measure
GRADE: Grading of Recommendations, Assessment, Development and Evaluation
INESSS: Institut national d'excellence en santé et services sociaux
KT: knowledge translation
PI: principal investigator
